# Structure–Function Relationships of Unusual Fatty Acids with Distinctive Functional Group Modifications and Carbon Chain Skeletons: Focus on the Roles of Double Bonds and Methyl Branches in Metabolism and Signaling

**DOI:** 10.3390/biology15151243

**Published:** 2026-07-28

**Authors:** Yuying Li, Lin Luo, Kai Song, Xiufang Huang, Mu Peng, Fang Chen, Zhiyong Wang

**Affiliations:** 1 Hubei Key Laboratory of Biological Resources Protection and Utilization, Hubei Minzu University, Enshi 445000, China; liyuying21103@163.com (Y.L.); luolin0430@163.com (L.L.); 2015069@hbmzu.edu.cn (X.H.); pengmu1025@hotmail.com (M.P.); 2024322040039@stu.scu.edu.cn (F.C.); 2 College of Biological and Food Engineering, Hubei Minzu University, Enshi 445000, China; 3 State Key Laboratory of Microbial Metabolism, Joint International Research Laboratory of Metabolic and Developmental Sciences, School of Life Sciences and Biotechnology, Shanghai Jiao Tong University, Shanghai 200240, China; jdsk@sjtu.edu.cn; 4 College of Life Science, Sichuan University, Chengdu 610065, China

**Keywords:** structurally unusual fatty acids, double bonds, methyl branches, β-oxidation, DSF-family signals, quorum sensing

## Abstract

Structurally unusual fatty acids (FAs) are biomolecules that connect lipid metabolism, membrane adaptation, and cellular communication. Evidence indicates that FA structural features, including double-bond position, stereochemical configuration, and methyl branches, influence physicochemical properties, metabolic fate, and biological functions. However, these mechanisms have not been systematically integrated. This review summarizes the structural characteristics and biological functions of epoxy, hydroxy, cyclopropane, acetylenic, and conjugated fatty acids. Among these, we focus particularly on structure–function relationships, metabolism of FAs with double bonds in uncommon positions and methyl branches, as these characteristics affect their recognition by metabolic enzymes and metabolic stability. We discuss the metabolic features and biological roles of structurally unusual FAs, with particular emphasis on how structural features regulate unsaturated FA β-oxidation. We clarify how double bonds affect isomerases and reductases through spatial configuration and how methyl branches may alter enzymatic recognition and slow degradation through steric effects. In diffusible signal factor (DSF)-family quorum-sensing signals, double bonds and methyl branches may contribute to the stability and effective concentration of signaling molecules by influencing β-oxidation-related turnover, potentially affecting signal intensity and duration. Overall, this review highlights the role of FA structures in linking metabolism with signal transduction. Future studies integrating biochemical assays, genetic analyses, and multi-omics approaches are needed to elucidate these mechanisms and expand applications.

## Simple Summary

Fatty acids are important molecules that help cells store energy, build membranes, and communicate. Some fatty acids have unusual chemical structures, such as special chemical bonds or small side branches, and these features can change how the molecules behave in cells. The relationships between these structural features, how the molecules are broken down, and their biological roles remain unclear. This review discusses several types of unusual fatty acids and explains how their structures influence their stability, recognition by cellular proteins called enzymes, and roles in cell membranes and signaling. Particular attention is given to bacterial communication molecules derived from fatty acids, because their structures may affect how long signals remain active and the strength of bacterial communication. By bringing together current knowledge, this review helps explain how the shapes of unusual fatty acids influence metabolism and bacterial behavior. A better understanding of these processes may support future studies on how microorganisms adapt to their environments, cell communication, and the development of useful fatty acid-based applications.

## 1. Introduction

Fatty acids are not only important substrates for energy metabolism and building blocks of membrane lipids, but also chemically diverse molecules whose biological functions are strongly influenced by structural features. Beyond common straight-chain fatty acids such as palmitic acid, stearic acid, oleic acid, linoleic acid, and linolenic acid [1], many naturally occurring fatty acids contain distinctive structural modifications, including unusual double-bond positions, *cis*/*trans* configurations, methyl branches, hydroxyl groups, epoxy groups, cyclopropane rings, or conjugated systems. These structural features can alter molecular conformation, polarity, membrane behavior, enzymatic recognition, and metabolic stability, thereby shaping their metabolic behavior and biological activities [2,3].

Compared with the relatively conserved fatty acid metabolism of higher plants, microorganisms have evolved diverse biosynthetic pathways for structurally unusual fatty acids during long-term adaptation to diverse or stressful environments and ecological interactions, thereby producing a variety of lipid molecules with unique chemical structures and biological functions [4]. These unusual fatty acids not only participate in the structural regulation of microbial cell membranes and environmental adaptation but also act as secondary metabolites involved in chemical defense and signal transduction. Fatty acids have long been regarded as fundamental substrates for microbial energy metabolism and membrane lipid biosynthesis; however, with advances in the study of metabolic networks and signal transduction, their roles as information-bearing molecules have received increasing attention. Rather than being defined solely by carbon-chain length, the functions of fatty acids are also critically determined by structural features such as double-bond position, *cis*/*trans* configuration, and methyl branching, which shape their metabolic fate and biological activities. In microorganisms, fatty acid β-oxidation is not only a central pathway for carbon-source utilization but also closely associated with pathogenicity, quorum sensing, and environmental adaptation. In particular, in the genus *Xanthomonas*, RpfB-dependent fatty acid activation and β-oxidation-associated pathways participate in the degradation and turnover of DSF-family quorum-sensing signals, thereby influencing virulence-factor expression and pathogenicity [5]. This phenomenon provides a useful model for understanding the intrinsic relationship among fatty acid structure, metabolic fate, and signaling function.

**Figure 1:**
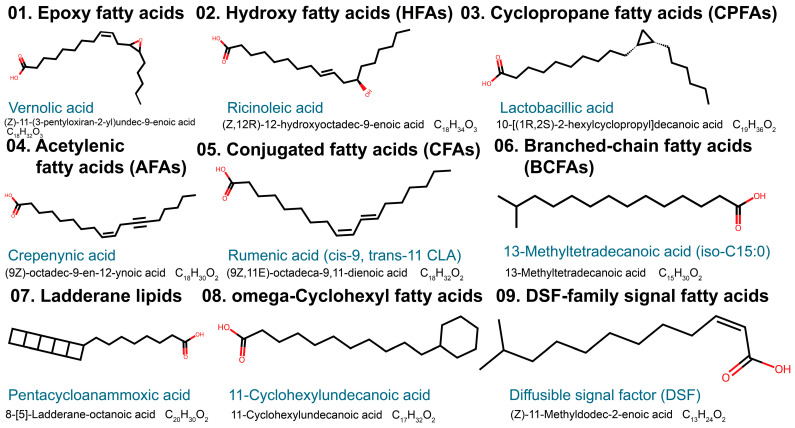
Representative structures of structurally unusual fatty acids. The selected compounds illustrate major structural motifs, including epoxy and hydroxy substitutions, cyclopropane and ladderane ring systems, carbon–carbon triple bonds, conjugated double bonds, methyl branching, terminal cyclohexyl groups, and distinctive unsaturation patterns. These compounds are presented as representative examples rather than universal structural skeletons for each fatty acid class.

To clarify the conceptual organization of this review, we discuss unusual fatty acids from three connected perspectives: structural motifs, metabolic processing, and biological functions. First, we summarize representative structural features, including functional-group modifications, ring systems, double-bond arrangements, and methyl branches. Second, we examine how these features influence physicochemical properties, enzymatic recognition, β-oxidation, lipid peroxidation, and metabolic turnover. Third, we link these metabolic consequences to biological functions, including membrane adaptation, stress responses, inflammatory injury, and bacterial communication. Overall, this review moves from structural classification to structure-dependent metabolic mechanisms, and finally to DSF-family signals as an example of fatty acid-derived molecules that connect structure, metabolism, and signaling. Representative compounds illustrating the major structural motifs discussed in this review are shown in Figure 1.

## 2. Research Background Based on Bibliometric Mapping

The bibliometric dataset was retrieved from the Web of Science Core Collection. Publications from 2000 to 2025 were searched using topic terms related to fatty acids, fatty acid metabolism, β-oxidation, structural features, and biological functions. The search strategy was set as TS = (“fatty acid” AND (“β-oxidation” OR “beta-oxidation” OR “quorum sensing”)). The search query was used to retrieve the bibliometric dataset. Keyword co-occurrence and burst analyses were then performed based on terms extracted from the retained records, including author keywords, Keywords Plus, titles, and abstracts. Only English-language articles and reviews were included, whereas meeting abstracts, editorial materials, book chapters, and duplicate records were excluded. The retained records were exported as full records with cited references. CiteSpace 6.4.R1 and VOSviewer 1.6.20 were used to analyze keyword co-occurrence, citation bursts, clustering timelines, and knowledge-network topology [6].

**Figure 2:**
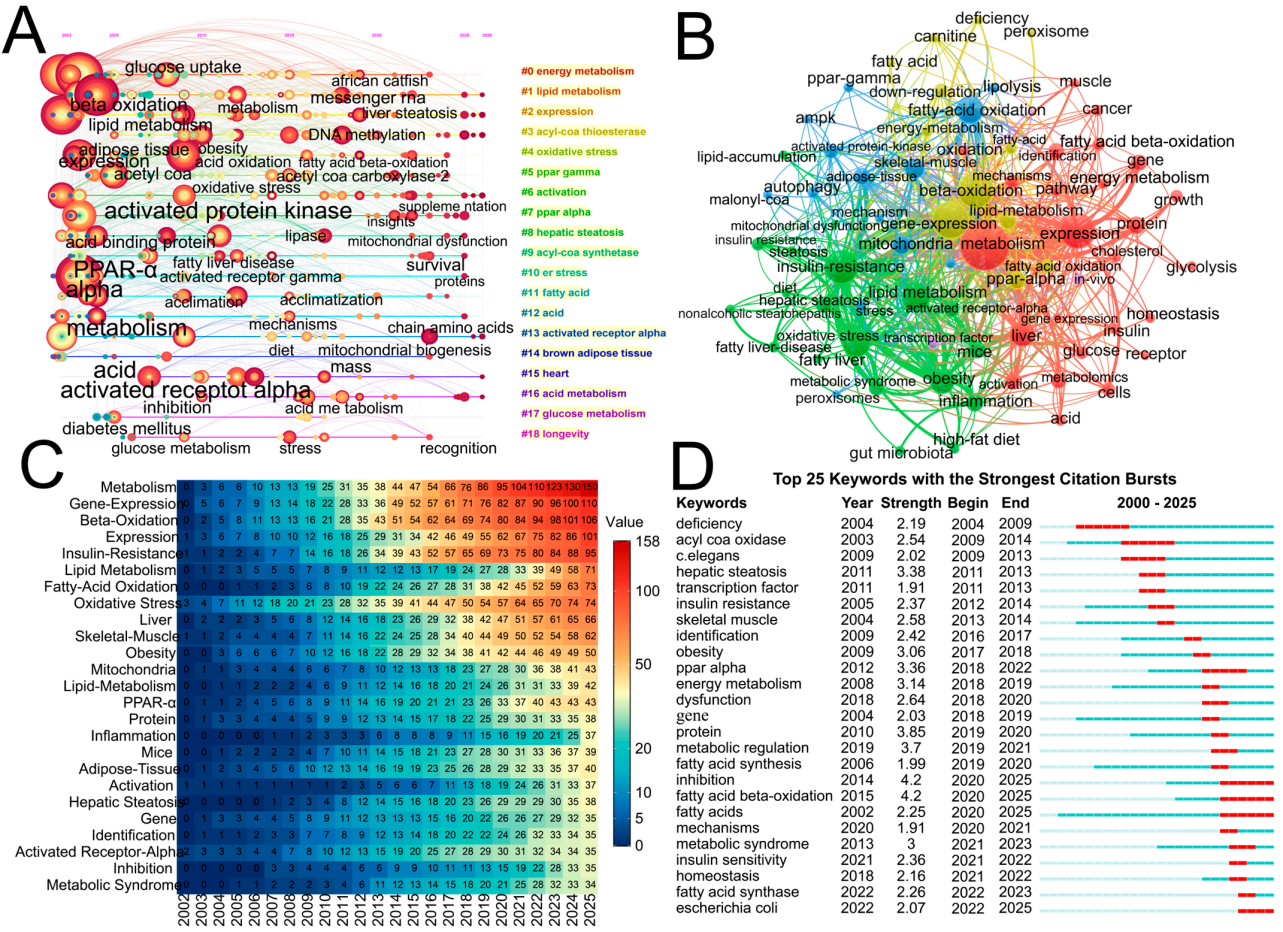
Bibliometric landscape linking fatty acid metabolism, β-oxidation, and signaling functions from 2000 to 2025. The bibliometric dataset was retrieved from the Web of Science Core Collection using the topic query TS = (“fatty acid*” AND (“β-oxidation” OR “beta-oxidation” OR “quorum sensing”)). (**A**) Keyword-clustering timeline (**B**) Keyword co-occurrence network (**C**) Keyword co-occurrence heatmap (**D**) Keyword bursts identified from the retained records.

The bibliometric analysis showed that, from 2000 to 2025, fatty acid research gradually shifted from broad metabolic studies to more focused investigations of molecular regulatory mechanisms. The timeline map (Figure 2A) indicates an early focus on physiological metabolism, followed by increasing attention to β-oxidation, peroxisomal metabolism, and transcriptional regulation. Keyword burst analysis (Figure 2B) further indicated that topics such as “fatty acid β-oxidation,” “inhibition,” and “metabolic regulation” have continued to receive sustained attention in recent years, suggesting that research hotspots have expanded from fatty acid synthesis and degradation to broader metabolic regulatory networks. The cumulative trends of high-frequency keywords (Figure 2C) and co-occurrence network analysis (Figure 2D) showed increasingly close connections among research directions related to metabolism, gene expression, oxidative stress, and cellular responses, thereby forming a knowledge framework centered on fatty acid metabolism and integrated with multiple layers of biological function. These results suggest that fatty acids are increasingly recognized not only as energy substrates but also as regulators of metabolic adaptation and cellular signaling.

Taken together, these bibliometric trends define the scope and sequence of this review. The sustained attention to β-oxidation, inhibition, and metabolic regulation is addressed in Section 4 through the discussion of how double-bond position and configuration affect fatty acid degradation. The recurrence of microbial metabolism, oxidative stress, cellular responses, and membrane-related terms is reflected in Section 3.2 and Section 5, which focus on microbial-derived unusual fatty acids and methyl-branched fatty acids. The emergence of quorum-sensing-related terms further motivates the discussion of DSF-family signals in Section 6 as representative fatty acid-derived communication molecules. Accordingly, the review proceeds from structural classification to structure-dependent metabolic processing and fatty acid-derived signaling systems.

## 3. Distinctive Structural Features and Biological Functions of Representative Fatty Acids

### 3.1. General Structural Characteristics of Representative Structurally Unusual Fatty Acid Classes

In addition to common straight-chain fatty acids, such as palmitic acid and oleic acid, nature contains a wide variety of structurally unusual fatty acids characterized by functional-group modifications, ring-containing structures, distinctive unsaturation patterns, or branched carbon-chain skeletons. Their designation as structurally unusual is based mainly on noncanonical structural motifs and related metabolic features, rather than solely on rarity in nature. Epoxy, hydroxy, cyclopropane, acetylenic, and conjugated fatty acids serve as representative examples of these structural deviations. Branched-chain fatty acids are also structurally unusual; the presence and position of methyl branches further link carbon-chain architecture with metabolic processing and fatty acid-derived signaling. The distinctive structural features, representative compounds, major sources, and biological functions or application values of these fatty acid classes are summarized in Table 1.

#### 3.1.1. Epoxy Fatty Acids

Epoxy fatty acid methyl esters are structurally modified lipid derivatives by epoxidizing the carbon–carbon double bonds (C=C) of unsaturated fatty acid methyl esters. This conversion changes the molecular conformation and electronic distribution, giving these compounds distinctive physicochemical properties [7]. Epoxidized fatty acid methyl esters generally dissolve well in various organic solvents and are compatible with polymer systems, facilitating their uniform dispersion in practical formulations. Their relatively low volatility also improves material stability and reduces losses caused by volatilization. Chemically, the epoxy group is the main reactive site and can undergo ring-opening reactions with compounds containing active hydrogen atoms, providing a basis for further functional modification [8,9]. Because of these properties, epoxidized fatty acid methyl esters have been widely explored as bio-based plasticizers, lubricant additives, and polymer modifiers.

#### 3.1.2. Hydroxy Fatty Acids

Hydroxy fatty acids (HFAs) are saturated or unsaturated fatty acids that contain one or more hydroxyl groups in their molecular structures. They occur naturally in animals, plants, and microorganisms, typically as components of cerebrosides, triacylglycerols, wax esters, and other lipid species [10,11,12]. Owing to hydroxyl substitution, HFAs generally have higher melting and boiling points, greater viscosity, and stronger chemical reactivity than many conventional fatty acids. Some HFAs have also been reported to exhibit biological activities, including antibacterial and anticancer effects. These structural and functional properties make HFAs valuable for applications in the chemical industry, cosmetics, pharmaceuticals, and biofuel-related materials [10,13].

#### 3.1.3. Cyclopropane Fatty Acids

Cyclopropane fatty acids (CpFAs) are a structurally distinctive group of saturated fatty acids that have been known from bacterial cell membranes for decades but have received relatively limited attention in metabolic studies. These molecules are synthesized by cyclopropane fatty acid synthase (CfaS), which catalyzes the conversion of double bonds in monounsaturated fatty acids (MUFAs) into three-membered cyclopropane rings. This reaction generates lipid derivatives with both the chemical stability of saturated fatty acids and the conformational constraints imposed by a cyclic structure. In recent years, CpFAs have attracted increasing interest as molecular markers for silage feeding in dairy authentication [14]. Studies have shown that when dairy cows are fed silage-containing diets, detectable levels of CpFAs accumulate in milk fat, particularly dihydrosterculic acid (DHSA) and lactobacillic acid (LBA) [15]. CpFAs also play important roles in regulating the physical and biological properties of bacterial cell membranes, including membrane fluidity, stability, proton permeability, growth adaptation, and acid tolerance [16].

Their association with bacterial stress tolerance, antibiotic resistance, and pathogenicity further suggests that CpFAs may provide useful clues for understanding bacterial membrane adaptation and for developing new antibacterial strategies [17,18,19]. This possibility is supported by evidence from *Helicobacter pylori*, in which deletion of cyclopropane fatty acid synthase reduced acid and antibiotic resistance, weakened immune evasion, and impaired gastric colonization. Pharmacological inhibition of CfaS activity further supports this enzyme as a potential antibacterial target [17]. A recent review also summarized the involvement of the *cfa* gene and CFAs in membrane permeability, membrane fluidity, membrane stability, antibiotic resistance, and bacterial pathogenicity [19]. These findings suggest that targeting CpFA biosynthesis may weaken bacterial stress adaptation and increase susceptibility to antibacterial treatments. Nevertheless, further studies are needed to evaluate the feasibility of this strategy across different pathogens and infection models.

#### 3.1.4. Acetylenic Fatty Acids

Acetylenic fatty acids (AFAs) are aliphatic fatty acids characterized by one or more carbon–carbon triple bonds (C≡C). Acetylenic lipids include a broad range of derivatives, such as alcohols, aldehydes, ketones, hydrocarbons, and fatty acids, and are widely distributed in nature [2,20]. Although most of these compounds occur at relatively low levels as secondary metabolites, they are associated with diverse biological functions. Some acetylenic fatty acids serve as major lipid components in the seed oils of certain higher plants, and related compounds have also been detected in bryophytes, fungi, algae, basal marine animals such as sponges, and some insects and bacteria. In producing organisms, these compounds often contribute to chemical defense, while some naturally occurring AFAs show pharmacological potential, including antibacterial and anticancer activities. Owing to the presence of triple bonds, acetylenic fatty acids are highly reactive and chemically unstable, especially under oxygen exposure. This chemical reactivity makes them useful for structural modification and functional exploration, but it also requires careful control of their isolation, storage, and application conditions [21,22].

#### 3.1.5. Conjugated Fatty Acids

Conjugated fatty acids (CFAs) are polyunsaturated fatty acids characterized by conjugated double-bond systems, in which adjacent double bonds are separated by a single carbon–carbon bond, forming a –C=C–C=C– structure [23]. Based on carbon-chain length, the number of double bonds, and geometric configuration, CFAs are generally classified into two major groups: conjugated linoleic acid (CLA), which comprises conjugated isomers of octadecadienoic acid (C18:2), and conjugated linolenic acid (CLNA), which comprises conjugated isomers of octadecatrienoic acid (C18:3). Because of their distinct double-bond arrangements, CLA and CLNA have attracted sustained interest in lipid chemistry and nutrition. These compounds have been reported to be associated with lipid metabolism, insulin sensitivity, platelet function and blood coagulation, tumor-related pathways, atherosclerosis, blood lipid regulation, immune responses, and bone metabolism [24,25,26,27]. Among these reported effects, the influence of CLA supplementation on body composition, particularly its possible association with increased lean mass and reduced fat mass, has been studied most extensively [28,29].

Plant seed oils are important natural sources of structurally unusual fatty acids. α-Eleostearic acid, a conjugated trienoic acid abundant in tung oil, is a representative plant-derived conjugated fatty acid whose conjugated double-bond system contributes to oxidative polymerization and drying-oil properties [30]. Vernolic acid, an epoxy fatty acid enriched in *Vernonia galamensis* seed oil, provides another example in which an epoxy group gives seed storage oils distinctive chemical properties and industrial relevance. These plant-derived fatty acids further illustrate how specific structural motifs, such as conjugated double bonds and epoxy groups, are linked to lipid accumulation, chemical reactivity, and practical applications [3,31]. Together, these examples outline major structural motifs of unusual fatty acids and provide a basis for the subsequent discussion of their microbial occurrence, ecological significance, and metabolism-related functions.

### 3.2. Microbial Diversity and Ecological Significance of Structurally Unusual Fatty Acids

Building on the structural diversity described above, microbial-derived unusual fatty acids can be further considered from the perspective of ecological function and adaptive value. Many microorganisms produce or remodel these lipids in response to environmental stress, host interactions, and interspecies competition. These fatty acids are therefore closely associated with membrane adaptation, stress tolerance, chemical defense, and signaling-related processes.

From the perspective of microbial function, structurally unusual fatty acids can be grouped according to their ecological and adaptive roles. CpFAs represent adaptive membrane lipids, and their accumulation is associated with acid resistance, antibiotic tolerance, and pathogenicity [19,32]. Branched-chain fatty acids are characteristic membrane components of many Gram-positive bacteria, particularly members of the genus *Bacillus*. They contribute to temperature-dependent membrane adaptation [19,33]. Polyacetylenic fatty acids from cyanobacteria and actinobacteria are often involved in chemical defense. For example, protegenins A–D produced by *Pseudomonas protegens* show inhibitory activity against fungal plant pathogens [34,35,36]. Lyngbyoic acid from the marine cyanobacterium *Lyngbya majuscula* can interfere with the quorum-sensing system of *Pseudomonas aeruginosa*. This feature suggests its potential relevance to anti-infective strategies [37,38]. Ladderane lipids in anammox bacteria support the formation of dense membrane barriers that restrict passive diffusion and help maintain energy metabolism [39,40,41]; ω-Cyclohexyl fatty acids in acidophilic and thermophilic bacteria, such as *Alicyclobacillus* spp., enhance membrane rigidity and stability under low-pH and high-temperature conditions [42,43,44].

Taken together, microbial fatty acids show a close link between structural diversity and biological function. Antifungal polyacetylenic fatty acids from actinobacteria, CpFAs from lactic acid bacteria used as markers of silage feeding, branched-chain fatty acids enriched in *Bacillus* spp., and ladderane lipids from anammox bacteria all illustrate how specific fatty acid structures support microbial adaptation and function. These molecules are relevant to extreme-environment adaptation, chemical ecological interactions, antibiotic resistance, and food traceability. Therefore, further clarification of the structural features, biosynthetic mechanisms, and physiological and ecological functions of microbial structurally unusual fatty acids will help explain the molecular basis of microbial adaptation and evolution. It may also provide useful lipid-based templates for developing biomaterials, drug leads, and biofuel-related applications. The structural classes described above indicate that unusual fatty acids differ not only in functional-group composition and carbon-chain architecture, but also in their interactions with metabolic enzymes. Among these structural determinants, the position and configuration of double bonds are particularly important because they can affect whether fatty acid intermediates can proceed through the conventional β-oxidation cycle. The following section therefore examines how patterns of unsaturation influence β-oxidation, auxiliary enzymatic requirements, and downstream metabolic consequences.

**Table 1:** Structural features and functional properties of representative structurally unusual and microbial-derived fatty acids.

Category	Distinctive Structural Features	Representative Fatty Acids	Major Sources	Biological Functions/ Application Value	References
Epoxy fatty acids	Carbon–carbon double bonds (C=C) are oxidized to form epoxy groups, resulting in a three-membered oxirane ring structure (–C–O–C–)	Epoxidized FAMEs	Vegetable oil derivatives, biodiesel processing products, yeasts, *Aspergillus* spp.	High reactivity through ring-opening reactions, good solubility and low volatility; used as plasticizers, lubricants, and coating additives	[9,45,46,47]
Hydroxy fatty acids (HFAs)	One or more hydroxyl groups (–OH) are introduced into the carbon chain	Ricinoleic acid and related HFAs	Plants, especially castor oil, animals, and intestinal lactic acid bacteria	Increased polarity and viscosity; antibacterial and anticancer activities; used in cosmetics, medicine, and biofuel-related applications	[13,48]
Cyclopropane fatty acids (CpFAs)	Carbon–carbon double bonds are converted into three-membered cyclopropane rings	Dihydrosterculic acid and lactobacillic acid	Bifidobacteria, *Escherichia coli* , lactic acid bacteria, and ruminant milk fat	Regulation of membrane fluidity and acid resistance; associated with antibiotic resistance and pathogenicity; molecular markers for dairy product traceability	[19,49,50]
Polyacetylenic fatty acids (AFAs)	Contain carbon–carbon triple bonds (C≡C), often including conjugated polyynes	Polyacetylenic fatty acids such as crepenynic acid and related compounds	Streptomyces spp., cyanobacteria, fungi, algae, and marine organisms	High reactivity; antibacterial, antifungal, and anticancer activities; potential for agricultural biocontrol and drug discovery	[21,51,52]
Conjugated fatty acids (CFAs)	Conjugated double-bond system (–C=C–C=C–)	Conjugated linoleic acid (CLA) and conjugated linolenic acid (CLNA)	Ruminant fat, dairy products, lactic acid bacteria, bifidobacteria, and *Bifidobacterium dentium*	Regulation of lipid metabolism; anti-atherosclerotic, anticancer, and immunomodulatory activities	[53,54]
Branched-chain fatty acids (BCFAs)	Carbon chains containing methyl branches, mainly iso- and anteiso-type structures	iso-C15:0 and anteiso-C17:0	*Bacillus subtilis*, *Bacillus cereus*, other Gram-positive bacteria, and *Listeria monocytogenes*	Maintenance of membrane fluidity and enhancement of temperature adaptability	[55,56,57,58]
Ladderane lipids	Multiple linearly fused cyclobutane rings forming ladder-like structures	Ladderane lipids	Anammox bacteria	Construction of highly dense membrane barriers; restriction of proton diffusion; maintenance of energy metabolism	[39,59,60]
ω-Cyclohexyl fatty acids(ω-CFAs)	Carbon chains containing a terminal cyclohexyl group	ω-cyclohexyl fatty acids	Acidophilic and thermophilic bacteria, such as *Alicyclobacillus* spp.	Enhanced membrane rigidity and stability; adaptation to high-temperature and low-pH environments	[42,61]
DSF-family signal fatty acids	*cis*-2-unsaturated fatty acids, usually with C10–C14 carbon chains; some contain methyl-branched chains or multiple double bonds	DSF, BDSF, CDSF, LeDSF	*Xanthomonas campestris* and related DSF-producing bacteria	Quorum-sensing signal molecules; regulation of virulence, biofilm formation, motility, and host interactions	[62,63,64,65]
“Signal-interfering” fatty acids	Structurally unusual fatty acid molecules, often containing unsaturation and/or modified functional groups	Lyngbyoic acid	Marine cyanobacterium *Lyngbya majuscula*	Inhibition of quorum sensing; potential value in anti-infective drug development	[37]

### 3.3. Structural Resolution of Fatty Acid Isomers in Lipid Analysis

Reliable interpretation of fatty acid structure depends on more than total fatty acid profiling. For fatty acids with different *cis/trans* configurations, unusual double-bond positions, conjugated systems, or methyl-branched chains, the separation and assignment of individual isomers are often critical. In GC-based lipid analysis, non-polar and mid-polar columns are useful for routine FAME profiling, but they usually do not provide sufficient resolution for closely related geometric or positional isomers. Detailed isomer analysis generally requires highly polar capillary columns, especially cyanopropyl stationary phases such as CP-Sil 88, SP-2560, DB-23, and Rtx-2330 [66]. Longer columns, commonly 60–100 m, are often used to improve the resolution of critical isomer pairs. Highly polar ionic-liquid stationary phases, such as SLB-IL111, can provide additional selectivity and have been applied to the separation of C18:1 and conjugated fatty acid isomers [66,67,68].

Reliable identification also depends on method validation. Column polarity and length alone do not ensure complete separation, especially in complex biological or food matrices. Separation efficiency should therefore be assessed with appropriate FAME reference mixtures or *cis/trans* isomer standards, particularly for isomer pairs that are prone to coelution. Official and widely used protocols, including AOAC 996.06, AOAC 2012.13, and AOCS Ce 1h-05, provide useful guidance for FAME preparation, chromatographic separation, and method evaluation [69]. Even with optimized chromatographic conditions, complete separation of all positional isomers is not always achievable. In such cases, authentic standards are essential for assigning individual isomers with confidence. When standards are not available, the results are better interpreted at a broader level, such as total trans isomers or unresolved isomer groups, rather than being over-assigned to specific positional isomers.

Temperature control also affects the resolution of fatty acid isomers. Low-temperature isothermal runs can improve the separation of some C18:1 positional isomers, whereas broader FAME profiles usually require gradient programs that balance resolution and analysis time. On a highly polar ionic-liquid column, an isothermal temperature of 120 °C has been shown to resolve all *cis*-C18:1 positional isomers and most *trans*-C18:1 positional isomers, although the critical *trans*-6-C18:1/*trans*-7-C18:1 pair required a higher temperature of 160 °C for separation [68]. Accurate assignment of these isomers is important for the discussion that follows, because double-bond position and configuration can determine whether unsaturated fatty acids proceed through the standard β-oxidation cycle or require auxiliary enzymatic steps.

## 4. Structure-Dependent Mechanisms Revealed by Unsaturated Fatty Acid β-Oxidation

### 4.1. Regulatory Effects of Double Bonds on the Basic Process of β-Oxidation

The frequent appearance of β-oxidation, inhibition, and metabolic regulation in recent studies highlights the importance of understanding how double-bond position and configuration affect fatty acid degradation. During the β-oxidation of saturated fatty acids, acyl-CoA dehydrogenase-mediated dehydrogenation is the typical first step. Each round of β-oxidation generates one FADH_2_ and one NADH, together with acetyl-CoA as an energy-producing product. FADH_2_ and NADH subsequently enter the electron transport chain and support oxidative phosphorylation and contribute to ATP generation [70]. In unsaturated fatty acids, however, pre-existing C=C double bonds modify the normal dehydrogenation sequence of β-oxidation. As a result, some dehydrogenation steps are bypassed, leading to reduced FADH_2_ production. This change affects energy output and also shows that double-bond position and configuration can influence the metabolic route of fatty acid degradation. To maintain β-oxidation, cells require auxiliary enzymes, such as Δ^3^–Δ^2^-enoyl-CoA isomerase and 2,4-dienoyl-CoA reductase, which convert noncanonical double-bond positions or configurations into substrates compatible with the standard β-oxidation cycle [71]. In most unsaturated fatty acids, double bonds occur in the cis configuration, and their positions often do not meet the substrate requirements of β-oxidation enzymes. Isomerases are therefore needed to shift the double bond to the C2–C3 (Δ^2^) position or to change its configuration, allowing the standard β-oxidation cycle to continue [72]. Specifically, 2,4-dienoyl-CoA reductase reduces conjugated 2,4-diene intermediates to trans-Δ^3^-enoyl-CoA in an NADPH-dependent manner, after which Δ^3^–Δ^2^-enoyl-CoA isomerase converts them into the canonical trans-Δ^2^ configuration. These reactions allow the intermediates to re-enter the conventional β-oxidation cycle. A previous peroxisomal study further showed that the NADPH required for this reduction can be supplied by isocitrate dehydrogenase, suggesting coordination between redox metabolism and the processing of unsaturated fatty acid intermediates. These findings indicate that the number, position, and conjugation state of double bonds are not passive structural features during β-oxidation. Instead, they determine whether auxiliary enzymes are required and whether fatty acids can be degraded smoothly or accumulate as pathway intermediates. Recent structural studies of β-oxidation enzymes have further shown that catalytic-site organization and substrate-binding cavities are closely related to chain-length preference, substrate specificity, and the coordination of consecutive β-oxidation reactions [73,74].

These findings suggest that β-oxidation is also involved in broader lipid metabolic regulation. Ding et al. [75] showed that peroxisomal β-oxidation contributes to lipolysis regulation in response to intracellular fatty acid availability. In this way, fatty acid oxidation may help coordinate fatty acid degradation, lipolytic activity, and lipid homeostasis [75].

### 4.2. Metabolic Adaptation Triggered by Atypical Double-Bond Positions

During β-oxidation, whether an unsaturated fatty acid can be continuously degraded is not determined only by its degree of unsaturation, but also by the position and configuration of its double bonds. The conventional β-oxidation system shows clear substrate selectivity and most efficiently processes trans-Δ^2^-enoyl-CoA intermediates [76]. When successive rounds of β-oxidation generate Δ^3^ (C_3_–C_4_) or other non-Δ^2^ double-bond intermediates, these compounds cannot be directly processed by the core β-oxidation enzymes. They therefore need to be converted into suitable intermediates by auxiliary enzymes, rather than simply entering the next round of degradation. This requirement explains why the β-oxidation of unsaturated fatty acids depends on isomerases and related auxiliary enzymes to overcome metabolic blockage caused by noncanonical double-bond positions [64,77]. Δ^3^-Enoyl-CoA isomerase, also known as Δ^3^→Δ^2^ enoyl-CoA isomerase or ECI1, is a key auxiliary enzyme in this process [78]. It converts Δ^3^-enoyl-CoA intermediates into the canonical trans-Δ^2^-enoyl-CoA form, allowing them to serve as substrates for β-oxidation hydratases. Early studies by Hiltunen et al. [79] in mammalian and peroxisomal systems showed that when the double bond is located near C_2_–C_3_ or C_3_–C_4_, ECI1 activity is required for β-oxidation to proceed; otherwise, metabolism stalls at the level of noncanonical double-bond intermediates. Subsequent molecular and biochemical studies have further shown that this isomerase-dependent route is conserved across different organisms. For example, van Weeghel et al. [80] demonstrated that mammalian mitochondria contain multiple Δ^3^/Δ^2^-enoyl-CoA isomerase isoenzymes with partially overlapping functions, all of which help redirect atypical double-bond intermediates into the standard β-oxidation pathway. These findings indicate that double-bond position, rather than the number of double bonds alone, is an important determinant of metabolic routing.

PUFA β-oxidation provides a clear example of this structure-dependent processing. Multiple double bonds increase the likelihood of forming noncanonical intermediates, such as conjugated 2,4-dienoyl-CoA species. These intermediates require auxiliary reduction and isomerization before re-entering the standard β-oxidation pathway [64,71]. Metabolic-network analyses further indicate that atypical double-bond positions introduce additional enzymatic steps and reshape the β-oxidation route [81]. Such adaptation may reduce immediate degradation efficiency, increase NADPH demand, and prolong the residence time of specific intermediates.

Thus, double-bond position helps determine whether unsaturated fatty acids can follow the standard β-oxidation cycle or require auxiliary processing. This mechanism helps explain why fatty acids with unusual double-bond patterns may differ in metabolic stability and regulatory potential.

### 4.3. Lipid Peroxidation of Membrane PUFAs and Formation of Reactive Aldehydes Under Inflammatory Oxidative Stress

In addition to serving as substrates for β-oxidation, polyunsaturated fatty acids (PUFAs) in membrane phospholipids are highly susceptible to lipid peroxidation under oxidative and inflammatory conditions. During inflammation, reactive oxygen species (ROS) are often produced at elevated levels. These reactive molecules can attack bis-allylic methylene groups in PUFAs and initiate lipid peroxidation [82]. This process is particularly relevant to omega-6 PUFAs, such as linoleic acid and arachidonic acid, and to omega-3 PUFAs, such as eicosapentaenoic acid and docosahexaenoic acid. The initial oxidation reactions generate lipid radicals and lipid hydroperoxides. These unstable intermediates can then break down into electrophilic reactive aldehydes [83].

Among these secondary products, 4-hydroxy-2-nonenal (4-HNE) is mainly formed from the peroxidation of omega-6 PUFAs, whereas 4-hydroxy-2-hexenal (4-HHE) is mainly generated from omega-3 PUFAs [82,84,85]. Unlike short-lived lipid radicals, these aldehydes are relatively stable. They can also diffuse away from their sites of formation, thereby extending the biological effects of lipid peroxidation. At low or moderate concentrations, 4-HNE and 4-HHE may participate in redox-related signaling [83,85]. However, during persistent inflammation or oxidative stress, their excessive accumulation can damage cells. This damage occurs mainly through covalent modification of proteins, phospholipids, and nucleic acids [82,83,85]. Such modifications may impair enzyme activity, disturb membrane function, and contribute to proteotoxic and genotoxic stress. Thus, the biological consequences of PUFA metabolism are not limited to enzymatic β-oxidation. The high degree of unsaturation in PUFAs also makes them vulnerable to oxidative fragmentation, linking fatty acid structure with inflammatory injury and lipid-derived signaling.

### 4.4. Stereochemical Issues Triggered by Double-Bond Configuration

During fatty acid β-oxidation, the configuration of double bonds affects not only pathway continuity but also the stereochemical form of the intermediates produced. In the conventional β-oxidation cycle, downstream enzymes, particularly 3-hydroxyacyl-CoA dehydrogenase, show strict stereochemical selectivity and generally recognize L-3-hydroxyacyl-CoA as the preferred substrate. However, the processing of unsaturated fatty acids with noncanonical double-bond positions or configurations may generate D-configured 3-hydroxyacyl-CoA intermediates, which cannot directly enter the standard β-oxidation cycle [86]. In such cases, 3-hydroxyacyl-CoA epimerase is required to convert the D-configuration into the L-configuration before the intermediate can undergo subsequent dehydrogenation and thiolytic cleavage [87]. This conversion may involve the coordinated action of enoyl-CoA hydratase-related enzymes, such as ECH1 and ECH2, and reflects the stereochemical constraints imposed by the β-oxidation enzyme system [87,88].

These stereochemical requirements further show that double-bond configuration is an important factor shaping fatty acid degradation routes. Unsaturated fatty acids may first require isomerization or reduction to place the double bond in a suitable position, and some resulting intermediates may need additional epimerization before they can be fully degraded. Thus, the stereochemical processing of unsaturated fatty acids is not simply a consequence of inefficient catalysis, but a specific enzymatic adjustment that allows structurally unusual substrates to re-enter the β-oxidation pathway.

### 4.5. Processing of Special Intermediates Derived from Polyunsaturated Fatty Acids

Because polyunsaturated fatty acids (PUFAs) contain multiple double bonds along their carbon chains, their β-oxidation can generate complex unsaturated intermediates. A typical example is 2,4-dienoyl-CoA, whose conjugated double-bond structure is not an efficient substrate for the core enzymes of the conventional β-oxidation cycle. Although auxiliary enzymes such as 2,4-dienoyl-CoA reductase and Δ^3^-enoyl-CoA isomerase allow these intermediates to be redirected into the standard β-oxidation pathway, their formation has broader metabolic consequences beyond pathway continuation [64,89].

First, the processing of PUFA-derived intermediates can alter cellular energy and redox balance. Compared with saturated fatty acids, PUFAs may bypass some dehydrogenation steps and require additional NADPH-dependent reduction reactions, leading to changes in FADH_2_ production and cofactor demand. Therefore, PUFA degradation is not only a carbon- and energy-yielding process, but also a redox-dependent metabolic route influenced by double-bond number, position, and configuration [75].

Second, the transient accumulation of PUFA-derived intermediates may influence lipid metabolic networks. Under conditions of altered metabolic load or environmental stress, noncanonical intermediates may persist for longer periods and interact with membrane lipid remodeling, stress-related signaling, or the formation of fatty acid-derived regulatory molecules [90,91]. This possibility is particularly relevant in microorganisms, where fatty acid metabolism is closely associated with environmental adaptation, pathogenicity, and quorum-sensing regulation.

**Figure 3:**
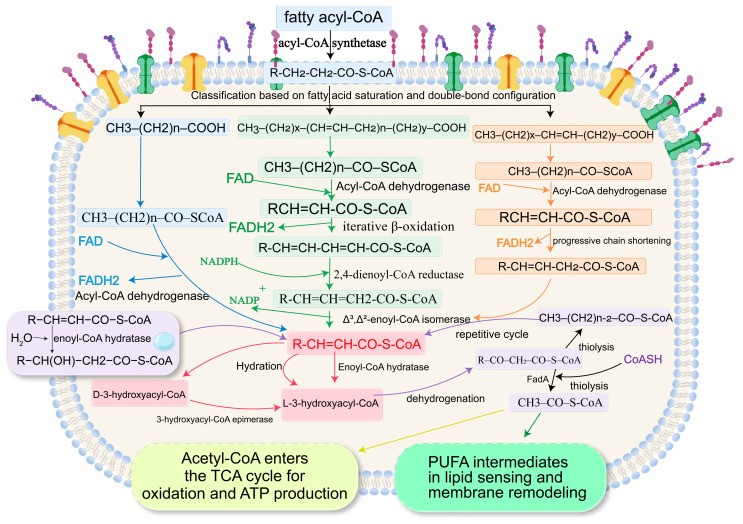
Intracellular β-oxidation pathways and divergence of functional fates of fatty acids with different chemical structures. The figure illustrates the differential β-oxidation routes of saturated fatty acids (blue), polyunsaturated fatty acids (green), and monounsaturated fatty acids (orange). Pink arrows indicate auxiliary processing steps for noncanonical unsaturated intermediates, including hydration, epimerization, and isomerization, whereas purple arrows indicate the conventional repetitive steps of β-oxidation. The central section shows the key auxiliary enzymatic steps required for processing noncanonical double-bond positions, including isomerization and stereochemical conversion during hydration reactions. The final section shows the possible metabolic fates of the resulting products, which may enter the tricarboxylic acid (TCA) cycle via acetyl-CoA for energy production or be retained as lipid intermediates associated with fatty acid sensing, stress responses, and membrane lipid remodeling.

Therefore, PUFA metabolism should be considered in relation to molecular structure and pathway-specific enzymatic processing, rather than being treated only as a general route for energy production [92]. The formation and turnover of PUFA-derived intermediates provide an important example of how double-bond architecture can influence not only β-oxidation efficiency but also downstream metabolic and regulatory functions. Compared with saturated fatty acids, mono- and polyunsaturated fatty acids may require auxiliary isomerization, reduction, or epimerization steps when pre-existing double bonds occur in noncanonical positions or configurations (Figure 3). However, unsaturation is only one structural determinant of metabolic fate. Methyl branching introduces another layer of structural variation that may affect enzyme recognition, degradation efficiency, membrane behavior, and signaling-related properties, as discussed below for methyl-branched fatty acids.

## 5. Metabolic Characteristics and Biological Significance of Methyl-Branched Fatty Acids

### 5.1. Structural Types and Distribution Characteristics of Methyl-Branched Fatty Acids

Methyl-branched fatty acids, commonly referred to as branched-chain fatty acids (BCFAs), represent a class of structurally unusual fatty acids in which the presence and position of methyl branches alter carbon-chain architecture and generate multiple structural isomers. These structural differences can influence membrane properties, enzymatic processing, and biological activity, making BCFAs useful examples for examining how carbon-chain architecture affects fatty acid function. The major types include iso-fatty acids, which have a near-terminal methyl group at the ω-1 position, and anteiso-fatty acids, which carry a methyl group at the ω-2 position. These structures often constitute a high molar proportion of microbial membrane fatty acids in microbial fatty acid profiles and may represent a substantial proportion of total cellular fatty acids. Common examples, including iso-C15:0, anteiso-C15:0, iso-C17:0, and anteiso-C17:0, illustrate the structural differences between iso- and anteiso-type BCFAs and often dominate membrane lipid composition [55,93]. Their formation mainly depends on the substrate specificity of branched-chain α-keto acid dehydrogenase complexes and related fatty acid synthases. In this pathway, branched-chain amino acid metabolites derived from isoleucine, leucine, and valine are converted into the corresponding branched-chain acyl-CoA primers. Thus, BCFA biosynthesis differs from straight-chain fatty acid biosynthesis mainly at the primer-selection step, leading to the formation of branch-containing fatty acid products [94].

In Gram-positive bacteria, BCFAs are major components of membrane lipids, particularly in species such as *Bacillus subtilis*, *Listeria monocytogenes*, and *Staphylococcus aureus* [95]. For example, in *Listeria*, BCFAs can account for most membrane fatty acids, with anteiso-type BCFAs, such as anteiso-C15:0 and anteiso-C17:0, often being dominant [57]. In contrast, Gram-negative bacteria such as *Escherichia coli* generally contain lower levels of BCFAs and more commonly regulate membrane properties through straight-chain saturated and unsaturated fatty acids. Pendleton and colleagues reported that BCFAs are required for the activation of multiple two-component systems in the Gram-positive bacterium *S. aureus* [58,96]. Beyond Gram-positive bacteria, BCFA enrichment has also been observed in other microorganisms, including certain plant pathogens and dairy-associated strains. For instance, lactic acid bacteria and related species such as *Propionibacterium freudenreichii* can synthesize and accumulate large amounts of iso- and anteiso-type BCFAs. These molecules function not only as major membrane lipid components but also as acyl donors in the N-fatty acylation of aromatic compounds, which may influence fermentation performance or host interactions in specific ecological niches [97].

Physiologically, BCFAs contribute to microbial membrane stability and environmental adaptation. Because methyl branches interfere with tight packing in lipid bilayers, BCFAs usually lower the melting point of membrane lipids and increase membrane fluidity and flexibility compared with straight-chain fatty acids [57,98]. This property is particularly important under environmental stresses such as temperature shifts and osmotic stress. The enrichment of BCFAs in the membranes of Gram-positive bacteria and some plant pathogens may therefore support colonization and stress adaptation. For example, the foodborne pathogen *L. monocytogenes* increases the proportion of anteiso-type BCFAs under low-temperature conditions, thereby lowering the membrane phase-transition temperature and improving membrane fluidity and cold tolerance. This response is consistent with homeoviscous adaptation, in which cells adjust membrane lipid composition to maintain an appropriate fluid state under environmental pressure [98].

Overall, methyl-branched fatty acids represent an important part of microbial fatty acid diversity. Their structural variation, including iso-, anteiso-, and more complex multi-methyl-branched forms, is closely linked to membrane biophysical properties and environmental adaptation. These features make BCFAs useful molecules for understanding microbial lipid adaptation, stress tolerance, and ecological fitness.

### 5.2. Effects of Methyl Substitution on β-Oxidation and Alternative Degradation Routes

The metabolism of methyl-branched fatty acids in the β-oxidation pathway differs from that of straight-chain fatty acids, mainly because methyl substitution changes substrate conformation and affects enzyme recognition [77,99,100]. Compared with straight-chain fatty acids, methyl branches cause the fatty acid backbone to deviate from a linear conformation, which may reduce the geometric fit between acyl-CoA substrates and the substrate-binding pockets of β-oxidation enzymes. As a result, acyl-CoA dehydrogenases, enoyl-CoA hydratases, and related enzymes may show altered affinity or catalytic efficiency toward branched substrates, although the extent of this effect depends on methyl-branch position, chain length, and enzyme specificity [101,102,103]. For example, some medium-chain 2-methyl fatty acids are oxidized less efficiently than their straight-chain analogs in mammalian systems [95,104]. These findings indicate that methyl-group position can influence substrate recognition and may limit conventional β-oxidation in a substrate-dependent manner [74,103,105].

For some branched-chain fatty acids, methyl substitution may require an alternative initial degradation route rather than simply reducing β-oxidation efficiency. Phytanic acid, a well-characterized 3-methyl-branched fatty acid, cannot directly enter conventional β-oxidation because of the methyl group at the β-carbon. In this case, peroxisomal α-oxidation first shortens the molecule by one carbon at the carboxyl end to generate pristanic acid, which can subsequently undergo β-oxidation. This pathway illustrates an important metabolic strategy for processing structurally branched substrates that are incompatible with the standard β-oxidation cycle [106,107].

In microbial systems, β-oxidation enzymes such as acyl-CoA dehydrogenase (FadE) and the multifunctional enzyme FadB are central to the degradation of straight-chain fatty acids [108]. When substrates contain methyl branches, however, their processing by these enzymes can become less efficient, depending on substrate structure and enzyme adaptation. Although FadE and FadB can efficiently catalyze reactions involving straight-chain fatty acids, branched-chain substrates may slower turnover, promote intermediate accumulation, or partially block the pathway [95,99]. In model bacteria such as *E. coli*, the β-oxidation system has a limited ability to process substrates with branched structures or irregular chain lengths, and auxiliary routes may be required for more complete degradation [109,110].

The ability to metabolize branched-chain fatty acids also varies among microorganisms, reflecting differences in enzyme structure, substrate range, and pathway regulation [57]. Some Gram-positive bacteria synthesize and accumulate large amounts of branched-chain fatty acids, suggesting that their lipid metabolic systems are adapted to branched substrates. In contrast, other bacteria appear to have a more limited capacity to degrade branched-chain fatty acids [111]. These differences may be related to the substrate specificity of core enzymes such as FadE and FadB, as well as to differences in the expression of auxiliary enzyme systems. In certain oleaginous microorganisms, β-oxidation is supported not only by conventional core enzymes but also by substrate-responsive binding modules and regulatory elements, allowing cells to use fatty acids from different environmental sources more flexibly [109,112]. These observations indicate that the effect of methyl branching on β-oxidation is not uniform, but depends on substrate structure, enzymatic specificity, and organismal metabolic capacity.

Overall, methyl branching can affect substrate conformation and interaction between fatty acyl-CoA substrates and β-oxidation enzymes, but its metabolic consequences are not uniform. Depending on methyl-branch position, chain length, enzyme specificity, and cellular context, methyl substitution may reduce degradation efficiency, require auxiliary pathways such as α-oxidation, or be partly accommodated by adapted β-oxidation systems. These differences suggest that β-oxidation systems have evolved different levels of flexibility in response to substrate diversity and ecological requirements.

### 5.3. Species Differences in Branched-Chain Fatty Acid Metabolism and Their Evolutionary Significance

The capacity to synthesize and degrade branched-chain fatty acids differs among microbial species. This variation is reflected not only in fatty acid synthesis and degradation pathways, but also in niche adaptation and metabolic regulation. Taking the Gram-positive genus *Bacillus* and the Gram-negative bacterium *Escherichia coli* as examples, these organisms differ in their use of branched-chain fatty acids, membrane lipid composition, and ecological adaptation.

In *Bacillus* species, branched-chain fatty acids are major components of membrane lipids. Iso- and anteiso-type BCFAs can account for large proportions of total fatty acids and strongly influence membrane properties, including fluidity and phase-transition behavior [97]. The abundance of these fatty acids helps *Bacillus* spp. maintain membrane stability under different temperature or chemical conditions and supports adaptation to stressful environments [113]. In contrast, Gram-negative bacteria such as *E. coli* mainly rely on straight-chain saturated and unsaturated fatty acids for membrane regulation. The β-oxidation system of *E. coli* primarily processes straight-chain substrates, whereas branched-chain fatty acids are present at low levels in its membrane and are not major membrane lipid components. This pattern reflects the distinct membrane-lipid strategy and metabolic organization of E. coli [114]. Although branched-chain fatty acid biosynthetic pathways can be introduced into *E. coli* through metabolic engineering, this capacity is limited in its native state, partly because its native fatty acid synthesis and degradation networks are not primarily configured for branched substrates than those of many Gram-positive bacteria [114,115].

From a functional perspective, branched-chain fatty acids can serve both as carbon and energy sources and as structural lipid components that regulate membrane properties. In ecological niches such as soil, hot springs, and extreme-temperature environments, BCFAs contribute to membrane stability and fluidity regulation, thereby improving microbial tolerance to environmental stress [33,116,117]. For example, *Bacillus* spp. can adjust the proportion of branched-chain fatty acids under cold or heat stress, helping maintain membrane function and cell viability. In addition, BCFAs may participate in stress-response and signaling-related processes, thereby influencing metabolic network adjustment and ecological fitness.

Overall, microbial fatty acid metabolism shows species-specific adaptation to environmental conditions and substrate availability. Some microorganisms use branched-chain fatty acids mainly as membrane components, whereas others have more limited capacity to synthesize or degrade them. The differences between *Bacillus* spp. and *E. coli* illustrate how fatty acid metabolic networks can be shaped by membrane requirements, carbon-source utilization, and ecological niche. These species-level differences provide a useful basis for understanding the functional diversification of microbial lipid metabolism.

### 5.4. Potential Functions of Methyl-Branched Fatty Acids in Signal Regulation

Fatty acids are increasingly recognized not only as substrates for energy metabolism but also as signaling-related molecules involved in cell–cell communication and environmental responses. For example, short-chain fatty acids can mediate intracellular signaling through G protein-coupled receptors, fatty acid transporters, and sensing proteins such as CD36, thereby influencing cellular metabolism and inflammatory responses [118]. Fatty acids differ in chain length, saturation, *cis/trans* configuration, and branching pattern, and these structural differences can affect their recognition by receptors and transporters. In animal systems, free fatty acid receptors (FFARs), a group of seven-transmembrane receptors, bind various fatty acids and their derivatives and participate in the regulation of energy homeostasis, immune responses, and metabolism-related gene expression. CD36 also functions as both a fatty acid transporter and a signaling-related sensor, linking fatty acid uptake with nutrient sensing and cellular regulation [119,120].

For methyl-branched fatty acids, the branched structure may lead to receptor- or transporter-interaction patterns that differ from those of straight-chain fatty acids. BCFA-rich lipid profiles can influence lipid transport, accumulation, and cellular fatty acid storage by affecting fatty acid transport proteins. These changes may not only alter metabolic pathways such as β-oxidation but also prolong the intracellular residence time of specific fatty acids, thereby influencing signal-related responses [121,122]. A study using *Caenorhabditis elegans* as a model showed that a BCFA-rich diet can inhibit β-oxidation and change the expression of lipid metabolism-related genes by affecting lipid droplet size and AMPK signaling activity. This suggests that branched-chain structures may influence cellular metabolic status by slowing fatty acid degradation and altering lipid signaling pathways [112]. Although most direct evidence currently comes from mammalian or nematode models, these findings provide a conceptual basis for discussing the possible roles of methyl-branched fatty acids in microbial fatty acid-based signaling [58].

In bacteria, emerging evidence suggests that BCFA-related lipid metabolism is also connected with membrane organization and signal-regulatory systems. For example, branched-chain amino acid metabolism controls membrane phospholipid structure in *Staphylococcus aureus*, and BCFAs are required for optimal activation of the Sae two-component system. BCFAs may also affect signal transduction indirectly by changing the membrane lipid environment. For example, 3-methyl and long-chain branched fatty acids can alter membrane fluidity, which may influence the localization and activation of fatty acid-sensing systems such as CD36 and FFAR4 [123,124]. In mammalian cell models, some short-chain fatty acids act as receptor agonists and activate downstream signaling pathways, supporting the idea that fatty acid structure can influence receptor recognition [125]. In bacterial quorum sensing, DSF-family fatty acid signals regulate downstream gene expression through dedicated sensing systems. This suggests that changes in fatty acid structure may affect signal stability, receptor affinity, and signal duration in quorum-sensing systems [126,127,128]. In addition to β-oxidation-dependent effects, fatty acid structure may also influence cellular responses through receptor-mediated sensing and oxidation-derived products. FFARs and CD36 link fatty acid recognition or uptake with energy homeostasis and metabolic gene regulation, whereas PUFA peroxidation generates reactive aldehydes, such as 4-HNE and 4-HHE, that participate in redox-related signaling under oxidative stress. These pathways are mechanistically distinct from β-oxidation, but they support the same principle that structural differences in fatty acids can reshape both metabolic status and signaling output.

Overall, methyl-branched fatty acids may influence signal regulation through several related mechanisms, including slower metabolic degradation, altered transporter recognition, changes in membrane properties, and modified receptor interactions. These effects suggest a possible connection between methyl branching, fatty acid metabolism, and microbial communication. However, direct evidence linking methyl-branched fatty acids to specific signaling pathways remains limited, and further biochemical and genetic studies are needed to clarify their roles in fatty acid-based quorum-sensing systems. Methyl-branched fatty acids illustrate how relatively small changes in carbon-chain architecture may influence metabolic processing and biological activity. This principle is also relevant to fatty acid-derived signaling molecules, where double bonds and methyl branches can affect signal stability, receptor recognition, and metabolic turnover. DSF-family quorum-sensing signals provide a representative example, linking fatty acid structure to metabolism-coupled bacterial communication.

## 6. Structural Features and Functional Mechanisms of Quorum-Sensing Fatty Acid Signals

### 6.1. Structural Features and Uniqueness of DSF-Family Quorum-Sensing Signals

Beyond fatty acid degradation and membrane adaptation, the emergence of quorum-sensing-related terms also points to the importance of fatty acid-derived molecules in bacterial communication. Many bacteria produce signaling molecules belonging to the diffusible signal factor (DSF) family. Most canonical DSF-family signals are medium-chain fatty acid-derived carboxylic acids containing a *cis*-2-unsaturated motif, whereas methyl substitution is variable rather than mandatory. Differences in chain length, additional double bonds, and methyl-branch position contribute to structural diversity and may affect receptor binding and signal recognition. DSF-family signals commonly regulate virulence-factor production, biofilm formation, antibiotic resistance, motility, and related phenotypes [65,129]. For example, *cis*-2-decenoic acid produced by *Pseudomonas aeruginosa* can induce biofilm dispersion and influence adhesion and collective behavior in several bacterial and fungal species [130]. DSF-like signals produced by *Xylella fastidiosa* include *cis*-2-tetradecenoic acid and *cis*-2-hexadecenoic acid, which regulate biofilm formation, motility, and pathogenicity-related gene expression in this species [131]. The Gram-positive bacterium *Streptococcus mutans* produces trans-2-decenoic acid, which inhibits the transition of *Candida albicans* from yeast to hyphal morphology, indicating that fatty acid signals can also participate in bacterial–fungal interactions [132]. Together, these findings show that DSF-family signals are structurally diverse and may function in both interspecies and inter-kingdom communication involving microorganisms, plants, insects, and animals [65,133,134].

DSF-family quorum-sensing signals were first characterized in the plant pathogen *Xanthomonas campestris* pv. *campestris*, with *cis*-11-methyl-2-dodecenoic acid (XcDSF) identified as a representative signal. Subsequently, several typical *cis*-2-unsaturated DSF-family signals were identified, including *cis*-2-dodecenoic acid (BDSF), *cis*,*cis*-11-methyldodeca-2,5-dienoic acid (CDSF), *cis*-10-methyl-2-dodecenoic acid (IDSF), *cis*-2-tetradecenoic acid (*Xf*DSF1), *cis*-2-hexadecenoic acid (*Xf*DSF2), and *cis*-2-decenoic acid (PDSF) [64]. The related signal 13-methyltetradecanoic acid, reported as LeDSF3, represents a structural exception to this *cis*-2-unsaturated framework [65]. Differences in chain length, methyl-branch position, and double-bond position may affect receptor recognition and signaling efficiency [135,136]. The core synthase RpfF belongs to the acyl-CoA dehydratase/thioesterase family and generates DSF-family signals from fatty acid biosynthetic intermediates. Thus, DSF signal formation is closely linked to fatty acid precursor structure and availability [136].

Overall, the *cis*-2-unsaturated fatty acid framework provides the core structural basis for most canonical DSF-family signals, whereas DSF-like or DSF-related molecules extend this concept to structurally related fatty acid signals. These features contribute to receptor recognition and signal perception, while also affecting the metabolic stability and biological activity of DSF molecules. Understanding these structural properties is therefore important for explaining how DSF-family signals regulate quorum sensing and how they interact with bacterial fatty acid metabolic networks.

### 6.2. Coupling Between the Biosynthesis, Degradation, and β-Oxidation Pathway of DSF-Family Quorum-Sensing Signals

In DSF-family quorum-sensing systems, signal biosynthesis, transport, and degradation are closely linked to fatty acid metabolism. RpfF converts 3-hydroxyacyl-ACP intermediates from fatty acid biosynthesis into *cis*-2-unsaturated DSF molecules through dehydration and thioesterase reactions [65,137]. After synthesis, the distribution and abundance of DSF-family signals inside and outside the cell are further affected by fatty acid transport and metabolic systems. RpfB activates DSF-family fatty acid signals and related medium-chain fatty acids to their CoA derivatives. This activation step is closely associated with DSF signal turnover and intracellular–extracellular homeostasis. In mutants lacking functional RpfB, extracellular DSF levels increase markedly, supporting the role of RpfB in DSF degradation and clearance [5,138,139]. Thus, DSF signaling is regulated not only by receptor perception but also by the metabolic fate of the signal molecule itself, which helps prevent excessive signal accumulation.

The β-oxidative degradation of DSF-family quorum-sensing signals provides a direct link between signal attenuation and fatty acid metabolism. The cis double bond and, in some DSF-family molecules, methyl-branched structures may influence enzymatic recognition during β-oxidation-associated turnover, thereby potentially affecting signal persistence and quorum-sensing duration [128]. In some DSF-like molecules, the pre-existing double bond may bypass the initial acyl-CoA dehydrogenase-catalyzed dehydrogenation step and alter subsequent enzymatic processing. Because cis-unsaturated structures are generally less favorable substrates for enoyl-CoA hydratase, hydration may become a limiting step in degradation. In addition, methyl branching may introduce steric hindrance and reduce the catalytic efficiency of downstream enzymes, such as L-3-hydroxyacyl-CoA dehydrogenase, by affecting enzyme–substrate binding [140,141]. However, this remains a plausible but insufficiently verified mechanism in DSF turnover, and direct biochemical evidence is still needed to confirm how methyl-branched DSF molecules are processed by β-oxidation enzymes. As DSF molecules are gradually degraded through β-oxidation, their effective concentration decreases, which may weaken quorum-sensing output and contribute to the transition of bacterial cells toward a basal physiological state [62].

### 6.3. A Unified Structure–Function View: DSF as a “Metabolism-Coupled” Signaling Molecule

DSF-family quorum-sensing signals are closely connected to bacterial fatty acid metabolism. Their production depends on fatty acid biosynthetic intermediates, their homeostasis is influenced by fatty acid transport and activation, and their attenuation is linked to β-oxidation. RpfF converts fatty acid intermediates into DSF-family signals through dehydratase and thioesterase activities, making DSF production a metabolic branch of fatty acid biosynthesis rather than an independent process [108,142]. This connection allows fatty acid metabolic status to influence quorum-sensing regulation [143,144].

Signal clearance is also tied to fatty acid metabolism. RpfB, a fatty acyl-CoA synthetase, converts DSF and related fatty acids into their CoA-bound forms, which can then enter downstream degradation pathways [5,145]. This activation step is therefore important for DSF turnover and signal attenuation. Homologues of RpfB in other species, such as *Bradyrhizobium japonicum*, have also been reported to participate in DSF degradation. Under iron-limited conditions, genes related to fatty acid metabolism can be induced, which may increase DSF degradation flux and connect signal turnover with environmental nutrient status [129]. These findings suggest that DSF degradation is not only a mechanism for terminating QS responses, but also part of a broader metabolic adjustment.

After activation, DSF degradation is closely associated with the β-oxidation pathway. The degradation rate of DSF-family signals depends partly on structural features such as double-bond position and methyl branching, which can affect recognition and processing by β-oxidation enzymes [5,127]. As a result, the intracellular residence time, effective concentration, and signaling duration of DSF molecules are shaped not only by receptor-binding kinetics but also by their metabolic fate. This structure-dependent processing provides a useful explanation for how DSF signals are attenuated after perception: signal attenuation is not separate from metabolism, but occurs through fatty acid transport, activation, and degradation.

**Figure 4:**
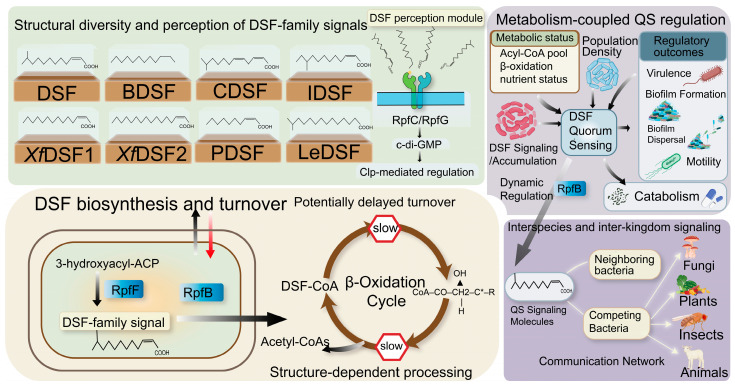
Structure-dependent turnover and regulation of DSF-family quorum-sensing signals. DSF-family and DSF-related molecules differ in acyl-chain length, double-bond arrangement, and methyl substitution, which may influence signal perception and metabolic turnover. The asterisk (*) indicates the stereogenic carbon center. RpfF generates DSF-family signals from fatty acid biosynthetic intermediates, whereas RpfB activates DSF and related fatty acids to CoA derivatives linked to β-oxidation-associated turnover. Signal perception through the RpfC/RpfG system regulates c-di-GMP and Clp-dependent transcriptional responses. Through these connected processes, DSF signaling links fatty acid metabolism with quorum-sensing outputs, including virulence, biofilm formation or dispersal, motility, antimicrobial tolerance, and interspecies or inter-kingdom interactions.

Overall, DSF-family signals provide a representative model of metabolism-coupled fatty acid signaling. Their biosynthesis, recognition, and turnover are linked to the fatty acid metabolic network, allowing bacterial cells to coordinate quorum sensing with nutrient status, environmental conditions, and physiological needs (Figure 4). In this framework, structural variation in DSF-family signals affects not only receptor recognition but also signal persistence through RpfB-associated activation and β-oxidation-associated turnover. More broadly, bacterial diffusible signaling molecules can regulate virulence, adhesion, biofilm formation and dispersion, motility, and antimicrobial tolerance, and they can also mediate interactions with fungi, plants, insects, and animals [143].

## 7. Conclusions and Perspectives

As organic compounds widely distributed in nature, fatty acids play important physiological roles in energy metabolism, membrane structure maintenance, and signal transduction. This review summarizes how structural features of fatty acids, including double-bond position and configuration as well as methyl branching, influence their metabolic processing and biological functions. The functions of fatty acids are not determined solely by chain length or degree of unsaturation. Instead, structural features such as double-bond position, double-bond configuration, and methyl branching can shape their roles in metabolic networks and further affect their activities as signaling molecules or regulatory factors. Overall, the reviewed evidence suggests that fatty acid structural features affect biological function through different but related mechanisms. Double-bond position and configuration can influence β-oxidation and PUFA oxidation, while methyl branching may affect enzyme recognition, degradation efficiency, and the turnover or recognition of some fatty acid-derived signals. This framework links the major topics discussed in this review, including unsaturated fatty acid β-oxidation, PUFA lipid peroxidation, methyl-branched fatty acid metabolism, and DSF-family signaling.

Progress in unsaturated fatty acid β-oxidation, methyl-branched fatty acid metabolism, and fatty acid-derived signaling molecules suggests that cellular metabolic systems can respond to fatty acid structural differences and translate them into distinct functional outcomes. Studies on unsaturated fatty acid β-oxidation show that double bonds do not simply reduce energy output. Rather, their position and stereochemical configuration can introduce additional enzymatic steps, such as isomerization and reduction, thereby altering reaction routes and intermediate homeostasis. This structure-dependent metabolic adaptation has been observed in different organisms, indicating that fatty acid structure is not merely a barrier to degradation but also an important factor affecting metabolic routing. Similarly, methyl-branched fatty acids often show lower degradation efficiency and more selective enzyme recognition because of conformational constraints. These features may extend the intracellular residence time of fatty acids and contribute to their potential roles in signal regulation.

Looking forward, several aspects of this research field require further investigation. First, future studies should clarify how structural features of fatty acid-derived signals influence their metabolic turnover, stability, and biological activity. Using DSF-family signals as a representative model, future studies could combine biochemical and enzymatic analyses to determine how structural features such as double-bond position and methyl substitution influence β-oxidation efficiency, signal stability, and biological activity. Second, genetic approaches, including the construction of RpfB, FadD, or other β-oxidation-related mutants, will help determine whether altered fatty acid activation and degradation directly affect DSF accumulation, turnover, and quorum-sensing output. Finally, metabolomic approaches, particularly stable-isotope tracing coupled with LC–MS/MS, could provide further insights into the metabolic fate of fatty acid signals and their regulatory roles. Together, these studies will help establish experimentally validated links between fatty acid structure, metabolic turnover, and signaling regulation.

## Data Availability

The original contributions presented in this study are included in the article. Further inquiries can be directed to the corresponding author.
